# Unintended costs and consequences of school closures implemented in preparation for Hurricane Isaac in Harrison County School District, Mississippi, August-September 2012

**DOI:** 10.1371/journal.pone.0184326

**Published:** 2017-11-01

**Authors:** Yenlik Zheteyeva, Jeanette J. Rainey, Hongjiang Gao, Evin U. Jacobson, Bishwa B. Adhikari, Jianrong Shi, Jonetta J. Mpofu, Darlene Bhavnani, Thomas Dobbs, Amra Uzicanin

**Affiliations:** 1 Division of Global Migration and Quarantine, US Centers for Disease Control and Prevention, Atlanta, Georgia, United States of America; 2 Office of Public Health Preparedness and Response, US Centers for Disease Control and Prevention, Atlanta, Georgia, United States of America; 3 Division of Preparedness and Emerging Infections, National Center for Emerging & Zoonotic Infectious Diseases, US Centers for Disease Control and Prevention, Atlanta, Georgia, United States of America; 4 Division of Reproductive Health, Chronic Disease Prevention and Promotion, US Centers for Disease Control and Prevention, Atlanta, Georgia, United States of America; 5 Epidemic Intelligence Service, Office of Workforce and Career Development, US Centers for Disease Control and Prevention, Atlanta, Georgia, United States of America; 6 Council of State and Territorial Epidemiologists Applied Epidemiology Fellowship, Atlanta, GA, United States of America; 7 Mississippi State Department of Health, Jackson, Mississippi, United States of America; Uniformed Services University of the Health Sciences, UNITED STATES

## Abstract

**Introduction:**

School closures, while an effective measure against the spread of disease during a pandemic, may carry unintended social and economic consequences for students and families. We evaluated these costs and consequences following a 4-day school closure in Mississippi’s Harrison County School District (HCSD).

**Methods:**

In a survey of all households with students enrolled in HCSD, we collected information on difficulties related to the school closure, including interruption of employment and pay, loss of access to subsidized school meals, and arrangement of alternative childcare. We analyzed this information in the context of certain demographic characteristics of the survey respondents and households, such as race, level of education, and income. We also estimated the average number of lost work days and documented the childcare alternatives chosen by households affected by the school closure.

**Results:**

We received 2,229 (28.4%) completed surveys from an estimated 7,851 households eligible to participate. About half (1,082 [48.5%]) of the households experienced at least some difficulty during the closure, primarily in three areas: uncertainty about duration of the closure, lost income, and the effort of arranging alternate childcare. Adults working outside the home, particularly the major wage earner in the household, were more likely to suffer lost income while schools were closed, an effect mitigated by paid leave benefits. Difficulty arranging childcare was reported most frequently by respondents with lower levels of education and households with younger children. Beyond the top three concerns expressed by households in HCSD, the survey also shed light on the issue of food insecurity when subsidized school meals are not available. Reported by 17.9% of households participating in the subsidized school lunch program, difficulty providing meals during the closure was associated with higher numbers of dependent children, selection of “other” as the race of the household respondent, and lower levels of education.

**Conclusion:**

To help prevent undue financial hardship in families of school children, public health authorities and school administrators should provide recommendations for childcare alternatives and paid leave or remote work options during prolonged school closures, particularly to households in which all adults work outside of the home.

## Introduction

School closures that are implemented timely and maintained for an appropriate duration, could reduce or delay transmission of influenza, lower the burden of illness on communities, and decrease morbidity and mortality during an influenza pandemic [1,2]. The US Centers for Disease Control and Prevention (CDC) therefore recommends pre-emptive, coordinated school closures as an important component of any strategy to mitigate a severe influenza pandemic [2], particularly in the initial stages when an effective vaccine is not available. Once implemented as a pandemic countermeasure, school closure could last up to several weeks depending on local epidemiology of the novel influenza virus [3].

Despite these potential benefits, however, unplanned school closures also raise social and economic concerns about parents missing work and losing pay in order to stay home with children, students missing subsidized school meals, and difficulties arranging and paying for alternative childcare [3]. A national survey suggested that compliance with community mitigation recommendations during a pandemic may be challenged if income or jobs were severely compromised; compliance would be particularly difficult for persons with lower income and for racial and ethnic minorities [4]. Similarly, some families may be disproportionately vulnerable to work absences during unplanned school closures, when they would have to stay home with children. Several studies [1] have examined the overall burden of missed work and lost pay on families during school closures, and found that the proportion of families in which an adult missed work can be as high as 28% [5,6]. However, there is little observational research into the factors that make some households more vulnerable to employment and pay interruptions than others.

Interruption of subsidized school meal service during school closures may affect 10–19% households, making the overall school closure experience difficult [5,7]. Eligibility for the free and reduced price meals program (run by the US Department of Agriculture) is determined annually based on the size of household and household income [8].

Better understanding of the unintended costs and consequences of unplanned school closures, and the population groups that may be most vulnerable, can help public health and education authorities plan for school closures during emergency situations [9]. While evidence for the effects of school closures in the specific context of an influenza pandemic is limited, certain prolonged school closures that occur during inter-pandemic years can serve as a proxy for evaluating their economic and social impact on student families. Seasonal influenza-related school closures rarely last longer than four days [9], and prolonged school closures during other emergency events can provide necessary insights.

On August 26, 2012, as Hurricane Isaac was predicted to make landfall, a state of emergency was declared in Mississippi and evacuation was recommended for residents along the coast and in some low-lying inland areas [10]. Nine coastal school districts in Mississippi were closed on Tuesday, August 28 to prepare for the anticipated hurricane and remained closed for the duration of the week (for a total of 4 days), while hurricane warning remained in effect. No major damages were made by the storm to school facilities [11].

The Mississippi State Department of Health requested CDC assistance in (1) evaluating the social and economic costs and consequences of unplanned school closures for student families, and (2) interviewing school officials about difficulties experienced by schools in Harrison County school district (HCSD), the largest of the nine districts that were closed to prepare for anticipated Hurricane Isaac.

## Materials and methods

CDC granted this project a non-research determination as an evaluation of the costs and consequences of unplanned school closures in the United States, supported by the Mississippi State Department of Health. No personally identifiable information was collected.

For the purpose of this evaluation, an unplanned school closure was defined as any instance in which a public or private school with any of the grades Kindergarten (K)-12 closes to all students for at least one school day not previously included as a closure in the school calendar. In the HCSD, the unplanned school closure that was the subject of this evaluation lasted from August 28-August 31, 2012 when all schools in the district were closed to students and staff.

### Study population

At the time of the 2012 school closure, HCSD was responsible for 20 schools, with 14,368 students enrolled in 12 elementary schools (6,517 students), two combined elementary and middle schools (2,557 students), three middle schools (1,663 students), and three high schools (3,631 students). The median percentage of students eligible for free or reduced-price school meals across the 20 HCSD schools was 73% (range, 45%-90%) [12]. In 2012, the majority (6770.9%) of the population in Harrison County was White, 22.13% was Black/African-American, 5.2% was Hispanic/Latino, 2.9% was Asian, and 2.87% was composed of other races/ethnicities. The average family size was 3.12, estimated median household income was $43,593.

### Data collection

We administered a household survey and conducted semi-structured interviews with school administrators in HCSD schools.

The survey questionnaire included questions regarding the demographic characteristics of each household, as well as perception of difficulties related to the school closure, childcare arrangements, interruptions in adult employment and income, missed subsidized school meals during the closure, estimated additional costs incurred for childcare, and communication of school closure information. We pilot-tested the survey prior to implementing the investigation in a small group of educators in order to ensure that the questions were appropriate and comprehensive.

We distributed paper questionnaires, along with a letter of support from the HCSD Superintendent, to all 14,368 HCSD students in November 2012. The letter provided instructions for completing the questionnaire and returning it to the schools within a week, as well as information on the consent process (completion and return of the survey implied consent). Assuming an estimated average of 1.83 school-aged children per household in Mississippi [14], an estimated 7,851 households were eligible for the survey. We requested that only one survey be completed per household (regardless of the number of children enrolled in district schools). The survey was anonymous and did not request any personal identifying information.

JM and DB conducted semi-structured interviews in person or over the phone with the principal, vice principal, or both at each participating school using a standard set of open-ended questions. Interviewers inquired about decision making and planning in this school closure; methods schools used to communicate school closure information to staff, students, and parents; the major challenges and issues faced by schools during the closures; and the strategies used to ensure continuation of school-based services during the school closures. Interview minutes were recorded in MS Word documents.

### Data management and analysis

Completed household surveys and interview responses from school officials were sent to the CDC headquarters in Atlanta, Georgia, for entry and analysis. Microsoft Access (Microsoft Corporation, Redmond, Washington, 2010) was used for data entry and management of the survey data. Data analysis was conducted in SAS v.9.3 (SAS Institute Inc., Cary, North Carolina) and STATA/SE v.12.1 (College Station, Texas). Information captured from the school administrator interviews was recorded in a Microsoft Word document.

We conducted univariate analyses of survey respondent and household demographics, types of difficulties caused by school closures, and communication channels used for announcing school closures. We evaluated factors, such as number of adults and children in household, adult employment, children’s age, primary respondent’s education, ethnicity and income for association with difficulty providing food as a result of subsidized school lunch program interruption, and difficulty arranging childcare. We also evaluated for association between gender, age, being parent/guardian, primary wage earner, employment location and work schedule with missing work or losing pay for all adult household members during the school closure.

For this evaluation, we used PROC GLIMMIX for mixed-effect univariate and multivariate logistic regression, which allowed us to account for potential correlations between observations. Variables that were significant at an alpha level of 0.10 in univariate analysis were included in multiple logistic regression models. An alpha level of 0.05 was used to assess statistical significance in multivariate analysis. We estimated the average number of lost work days for households that did and did not lose pay and for select household compositions. We evaluated childcare choices made by families during the school closure and examined these choices as a function of select household characteristics. We also calculated the cost to households of various childcare alternatives.

Minutes from school administrator interviews were reviewed and broken into topic categories within each interview question. Concepts and issues were included for consideration in the study when reported by school administrators in 4 or more interview sessions.

## Results

### Household survey

We received completed surveys from 2,229 households (response rate of 28.4%). The majority of respondents were White (1,434 [64.3%]), and more than half of the responding households (1,289 [57.9%]) reported an annual household income less than $50,000. The majority of respondents (1,378 [61.8%]) were members of two-adult households, and 536 (24.1%) were members of single-adult households. Most of the 4,247 adults worked full time (2,530 [59.6%]) and were employed outside of household (2,903 [68.3%]). In 396 (75%) of single-adult households, the adult worked outside of household, whereas in 754 (45.3%) household with two and more adults, all adults were employed outside of home. Slightly more than one-third (1,611 [37.9%]) of adults employed outside of the home received paid time off. Most of the 4,171 children in responding households were younger than 12 years of age (2,794 [67.8%]), and half (2,116 [50.7%]) were enrolled in elementary schools (grades 1–5) (Table 1).

**Table 1:** Demographic and economic characteristics of survey respondents and households. Unplanned School Closure Household Survey, Harrison County School District, Mississippi, August 2012 (N = 2,229).

Variable Name and Category	N (%)
**Respondent Race/Ethnicity**	
White, Non-Hispanic	1,434 (64.3)
Black/African-American, Non-Hispanic	548 (24.6)
Asian	82 (3.7)
Hispanic/Latino	47 (2.1)
Other*	80 (3.6)
Missing/refused to answer	38 (1.7)
**Respondent Education**	
High school or less	770 (34.5)
College: 1–3 years or technical school training	759 (34.1)
College: 4 years or more (college graduate)	374 (16.8)
Graduate or professional school (1 year or more)	196 (8.8)
Missing/refused to answer	130 (5.8)
**Household Income**	
< $25,000	710 (31.9)
$25,000-$49,999	579 (26.0)
$50,000 - $74,999	270 (12.1)
≥ $75,000	302 (13.5)
Missing/refused to answer	368 (16.5)
**Household Composition**	
Average Household Size (median; range) 3.8 (4; 2–10)	
Number of adults per household	
1 adult	536 (24.1)
2 adults	1,378 (61.8)
3 adults	222 (10.0)
4 adults	52 (2.3)
5 adults	14 (0.6)
Unknown	27 (1.2)
Single-adult households (n = 536),	
Adult employed outside of household	396 (75.0)
Households with 2 or more adults (n = 1,666)	
All adults employed outside of household	754 (45.3)
Number of children per household	
1 child	945 (42.4)
2 children	824 (36.9)
3 children	344 (15.4)
4 children	82 (3.7)
5 children	33 (1.5)
Unknown	1 (0.1)
**Adult characteristics (n = 4,247)**	
Adults sex, male	1,804 (42.9)
Average age of adults (SD), range	38.5 (11.1), 19–93
Adults employment status and schedule	
Full-time	2,530 (59.6)
Part-time	340 (8.0)
No fixed schedule	206 (4.9)
Not employed	1,000 (23.5)
Missing/refused to answer	171 (4.0)
Adults employed outside of home	2,901 (68.3)
Employed adult receives paid time off (sick and/or annual leave)	1,611 (37.9)
**Children characteristics (n = 4,171)**	
Children sex, male	2,017(48.4)
Children grade level^†^	
Head Start program, Pre-K and K	355 (8.5)
1–5	2,116 (50.7)
6–8	830 (19.9)
9–12	761 (18.2)
Unknown	109 (2.6)
Children under 12 years old^§^	2,794 (67.0)

* “Other” race includes households in which the survey respondent self-identified as Native Hawaiian/Pacific Islander or Native American Indian/Alaska Native, or checked “other” race, which included combinations of two or more races

^†^ Grade level reported for 4,062 children.

^§^ Child’s age in years is determined based on school grade level as follows: Head Start: 3, Pre-Kindergarten: 4, Kindergarten: 5, 1st grade: 6, 2nd grade: 7, 3rd grade: 8, 4th grade: 9, 5th grade: 10, 6th grade: 11, 7th grade: 12, 8th grade: 13, 9th grade: 14, 10th grade: 15, 11th grade: 16, 12th grade: 17–18.

Of the 2,229 households, about half (1,082 [48.5%]) reported that the unplanned school closure caused at least some difficulties (Table 2). If the schools were closed for 1 month, it would not be a problem for 1,021 (45.8%) respondents. Among 1,720 (77.2%) respondents who reported a child’s enrollment in the subsidized school lunch program, 308 (17.9%) experienced difficulty providing meals to their families due to interruption of the program during the school closure. Almost half of adults [1,793 (45.6%)] missed work during the school closure. The majority of them reported missing 1–2 days (815 [45.5%]), while 123 (6.9%) missed more than 1 week (Table 2). Households in which at least one adult lost pay missed, on average, 3.75 workdays, whereas households not losing pay missed only 1.21 workdays. There was variability in the number of missed workdays depending on household composition: single-adult households lost, on average, 2.38 days, whereas two-adult households lost 3.35 days (one working adult) and 4.3 days (both adults working), respectively (data not shown).

**Table 2:** Reported difficulties and employment and income interruptions related to unplanned school closure, Harrison County School District, Mississippi, August 2012 (N = 2,229).

Variable Name and Category	N (%)
**Parental perception of difficulty of school closure**	
Not difficult	1,099 (49.3)
At least some difficulty:*	1,082 (48.5)
Did not know how long school would be closed	677 (62.6)
Lost income due to missed work	599 (55.4)
Difficult to make childcare arrangements	314 (29.0)
Expensive to make childcare arrangements	194 (17.9)
Student missed school meals	144 (13.3)
Other	108 (10.0)
Missing/refused to answer	48 (2.2)
**If schools had to be closed for ONE MONTH, how big of a problem would it be?**	
Not a problem	1,021 (45.8)
Major problem	566 (25.4)
Moderate problem	239 (10.7)
Minor problem	173 (7.8)
Do not know/Not sure	196 (8.8)
Missing/refused to answer	34 (1.5)
**Difficulty in providing food due to lost access to free/reduced price lunch (n = 1,720)**	
No	1,412 (82.1)
Yes	308 (17.9)
**Interruption of adult employment and pay (n = 4,247)**	
Adults missed work during school closure	1,793 (45.6)
Adults lost pay during school closure	1,256 (29.6)
If adult missed work (n = 1,793), for how many days:	
1–2 days	815 (45.5)
3–5 days	779 (43.4)
> 1 week	123 (6.9)
Missing/refused to answer	76 (4.2)

* Responses are not mutually exclusive

Logistic regression models were used to further evaluate household and respondent characteristics associated with difficulty providing food and arranging childcare, as well as with adults in respondent households missing work or losing pay during this school closure.

When evaluating difficulty providing food as a result of lost access to subsidized school meals, households with three children (adjusted odds ratio [aOR], 1.68; 95% CI, 1.15–2.47) and those who selected “other” as the survey respondents’ race (aOR, 2.26; 95% CI, 1.17–4.36) were significantly more likely to report difficulty providing food, whereas households with an annual income of $50,000 or more were significantly less likely (aOR, 0.21; 95% CI, 0.12–0.36) to report such difficulty (Table 3).

**Table 3:** Factors associated with difficulty providing food due to lost access to subsidized school lunches during unplanned school closure. Harrison County School District, Mississippi, August 2012.

Variable Name	Variable Category	Univariate Analysis*		Multivariate Analysis**	
		OR(95% CI)	p-value	aOR(95% CI)	p-value
Number of adults in household					
	1	Ref	—	Ref	—
	2	0.64 (0.48–0.84)	0.001	0.90 (0.65–1.26)	0.552
	3	0.82 (0.53–1.56)	0.365	1.02 (0.61–1.72)	0.935
	4,5^§^	1.13 (0.58–2.20)	0.710	0.89 (0.41–1.95)	0.774
All adults employed outside of home	Yes vs. No	0.82 (0.64–1.05)	0.111	—	—
Number of children in household					
	1	Ref	—	Ref	—
	2	1.14 (0.85–1.53)	0.384	1.12 (0.80–1.56)	0.503
	3	1.87 (1.33–2.63)	< 0.001	1.68 (1.15–2.47)	0.007
	4,5^§^	2.00 (1.23–3.26)	0.005	1.52 (0.88–2.62)	0.131
If any child in household is older than 12 years old	Yes vs. No	1.04 (0.81–1.34)	0.738	—	—
Ethnicity of household survey respondent					
	White Non-Hispanic	Ref	—	Ref	—
	Black/African-American, Non-Hispanic	1.56 (1.18–2.05)	0.002	1.19 (0.86–1.64)	0.292
	Asian	0.94 (0.44–2.03)	0.874	1.07 (0.43–2.67)	0.890
	Hispanic/Latino	1.38 (0.59–3.21)	0.455	0.96 (0.35–2.64)	0.940
	Other^¶^	2.56 (1.45–4.52)	0.001	2.26 (1.17–4.36)	0.015
Education of survey respondent					
	Graduate or Professional School	Ref	—	Ref	—
	College	1.41 (0.72–2.76)	0.318	1.23 (0.58–2.62)	0.586
	Some College	2.12 (1.16–3.90)	0.015	1.39 (0.70–2.77)	0.345
	High school or less	2.56 (1.40–4.68)	0.002	1.55 (0.78–3.09)	0.212
Household Income					
	< $25,000	Ref	—	Ref	—
	$25,000-$49,999	0.73 (0.55–0.97)	0.031	0.78 (0.57–1.07)	0.121
	≥ $50,000†	0.16 (0.10–0.27)	< 0.001	0.21 (0.12–0.36)	< 0.001

OR: odds ratio, aOR: adjusted odds ratio, CI: confidence interval.

*Univariate logistic regression.

**Multiple logistic regression: only variables significant at alpha = 0.1 in univariate analysis were included.

^†^Household income ≥ $75,000 was combined with the $50,000-$74,999 category because of the low count of outcomes in these subgroups.

^§^Households with 4 or 5 adults were combined because of the low count. Households with 4 or 5 school children were combined as well because of the low count

^¶^“Other” race includes households in which the survey respondent self-identified as Native Hawaiian/Pacific Islander or Native American Indian/Alaska Native, or checked “other” race, which included combinations of two or more races

With regard to alternative childcare, households in which all adults were employed outside of the home (aOR, 3.16; 95% CI, 2.30–4.35) and households in which the survey respondent had some college or a college degree (aOR, 2.79 and 2.32; 95% CI, 1.52–5.10 and 1.23–4.36, respectively) were significantly more likely to report difficulty making arrangements. Households in which at least one child was older than 12 years of age and households with an annual income higher than $75,000 were similarly (about 50%) less likely to report difficulty arranging childcare during this school closure (aOR, 0.50 and 0.58; 95% CI, 0.38–0.65 and 0.36–0.94, respectively) (Table 4).

**Table 4:** Factors associated with difficulty arranging childcare during school closure. Harrison County School District, Mississippi, August 2012.

Variable Name	Variable Category	Univariate Analysis*		Multivariate Analysis**	
		OR(95% CI)	p-value	aOR(95% CI)	p-value
Number of adults in household	1	Ref	—	Ref	—
	2	0.51 (0.40–0.65)	< 0.001	0.74 (0.54–1.02)	0.066
	3	0.33 (0.20–0.53)	< 0.001	0.72 (0.40–1.29)	0.268
	4,5^§^	0.41 (0.19–0.89)	0.024	1.06 (0.44–2.53)	0.905
All adults employed outside of home	Yes vs. No	3.27 (2.51–4.25)	< 0.001	3.16 (2.30–4.35)	< 0.001
Number of children in household *	1	Ref	—	—	—
	2	0.94 (0.74–1.21)	0.642	—	—
	3	0.92 (0.66–1.28)	0.619	—	—
	4,5^§^	0.50 (0.26–1.52)	0.235	—	—
If any child in household is older than 12 years old	Yes vs. No	0.51 (0.41–0.65)	< 0.001	0.50 (0.38–0.65)	< 0.001
Ethnicity of household survey respondent*	White Non-Hispanic	Ref	—	—	—
	Black/African-American, Non-Hispanic	1.18 (0.91–1.54)	0.208	—	—
	Asian	1.22 (0.68–2.17)	0.508	—	—
	Hispanic/Latino	1.47 (0.72–3.00)	0.292	—	—
	Other^¶^	1.58 (0.92–2.72)	0.101	—	—
Education of primary caregiver/guardian	Graduate or Professional School	Ref	—	Ref	—
	College	2.16 (1.24–3.75)	0.006	2.32 (1.23–4.36)	0.009
	Some College	2.62 (1.57–4.39)	< 0.001	2.79 (1.52–5.10)	< 0.001
	High school or less	1.70 (1.01–2.87)	0.047	1.71 (0.91–3.21)	0.094
Household Income	< $25,000	Ref	—	Ref	—
	$25,000-$49,999	0.72 (0.54–0.96)	0.024	0.71 (0.51–0.99)	0.041
	$50,000-$74,999	0.84 (0.59–1.21)	0.347	0.69 (0.45–1.06)	0.086
	≥ $75,000	0.60 (0.41–0.87)	0.007	0.58 (0.36–0.94)	0.025

OR: odds ratio, aOR: adjusted odds ratio, CI: confidence interval.

*Univariate logistic regression.

**Multiple logistic regression: only variables significant at alpha = 0.1 in univariate analysis were included.

^§^Households with 4 or 5 adults were combined because of the low count. Households with 4 or 5 school children were combined as well because of the low count

^¶^ “Other” race includes households in which the survey respondent self-identified as Native Hawaiian/Pacific Islander or Native American Indian/Alaska Native, or checked “other” race, which included combinations of two or more races

While controlling for adults’ individual and employment characteristics, major household wage earner status (aOR, 1.42; 95% CI, 1.06–1.90) and employment outside the home (aOR, 2.78; 95% CI, 1.81–4.26) remained independently associated with missed work or lost pay. Adults who received paid time off were significantly less likely to miss work or lose pay (aOR, 0.57; 95% CI, 0.48–0.68) (Table 5).

**Table 5:** Factors associated with adult household members missing work or losing pay during unplanned school closure, Harrison County School District, Mississippi, August 2012.

Variable name	Variable Category	Univariate Analysis*		Multivariate Analysis**	
		OR(95% CI)	p-value	aOR(95% CI)	p-value
Sex	Female vs. Male	1.00(0.88–1.15)	0.95	—	—
Adult age (in years)	18–44	Ref	—	Ref	—
	45–64	0.84(0.71–1.00)	0.056	0.94(0.76–1.16)	0.540
	65+	0.13(0.06–0.25)	<0.001	0.65(0.22–1.93)	0.434
Adult is parent or guardian	Yes vs. No	1.61(1.28–2.03)	<0.001	0.94(0.67–1.32)	0.727
Adult is major wage earner in the household	Yes vs. No	4.25(3.57–5.06)	<0.001	1.42(1.06–1.90)	0.020
Adult is employed outside of the household	Yes vs. No	14.11(11.10–17.95)	<0.001	2.78(1.81–4.26)	<0.001
Work schedule	Full time	Ref	—	Ref	—
	No fixed work schedule	0.64(0.47–0.87)	0.005	0.81(0.54–1.22)	0.319
	Part-time	1.25(0.97–1.62)	0.091	1.14(0.84–1.55)	0.389
If adult received paid time off(annual/sick leave)	Yes vs. No	1.57(1.37–1.80)	<0.001	0.57(0.48–0.68)	<0.001

OR: odds ratio, aOR: adjusted odds ratio, CI: confidence interval.

*Univariate logistic regression.

**Multiple logistic regression after accounting for household cluster effect: model includes variables that were significant at alpha = 0.1 in univariate analysis.

Among all households, a non-working adult or an adult who works outside of home were the most frequent childcare providers during this school closure (868 [38.9%] and 467 [20.9%], respectively). For single-adult households in which the adult works outside of home, childcare was most frequently provided by an adult who does not live in the household (125 [31.6%]), while households with two or more adults who all work outside the home most frequently reported that a working adult stayed home with the children (264 [35%]) (Table 6).

**Table 6:** Frequency of using alternative childcare options by selected household characteristics. Harrison County School District, Mississippi, August 2012.

	Who provided childcare during the school closure in HCSD?								
	Adult household member who works outside the household	Adult who does not live in the household	Non-working adult household member	Took children to work	Older sibling	Childcare program	Child old enough to care for him/herself	Children left home without supervision	Adult working from home
**All households**									
**All households** **(N = 2,229)**	467 (20.9%)	332 (14.9%)	868 (38.9%)	118 (5.3%)	163 (7.3%)	58 (2.6%)	259 (11.6%)	56 (2.5%)	88 (3.9%)
**Households in which at least one adult stays home (n = 1,066)**	107 (10.0%)	64 (6.0%)	739 (69.3%)	27 (2.5%)	50 (4.7%)	15 (1.4%)	109 (10.2%)	14 (1.3%)	46 (4.3%)
**Households with income <25,000 (n = 710)**	103 (14.5%)	129 (18.2%)	322 (45.4%)	38 (5.4%)	55 (7.7%)	25 (3.5%)	67 (9.4%)	16 (2.3%)	28 (3.9%)
**Households with income≥75,000 (n = 302)**	101 (33.4%)	36 (11.9%)	66 (21.9%)	21 (6.9%)	24 (7.9)	4 (1.3%)	46 (15.2%)	10 (3.3%)	20 (6.6%)
**Single adult households**									
**All single adult households (n = 536)**	98 (18.3%)	139 (25.9%)	144 (26.9%)	32 (6.0%)	45 (8.4%)	21 (3.9%)	57 (10.6%)	13 (2.4%)	23 (4.3%)
**Single adult households in which adult is employed outside of home (n = 396)**	92 (23.2%)	125 (31.6%)	N/A	31 (7.8%)	40 (10.1%)	20 (5%)	43 (10.9%)	11 (2.9%)	13 (3.3%)
**Households with** **≥ 2 adults**									
**All ≥ 2 adult households (n = 1,666)**	364 (21.9%)	189 (11.3%)	716 (42.9%)	85 (5.1%)	117 (7.0%)	35 (2.1%)	196 (11.8%)	43 (2.58)	65 (3.9)
**≥ 2 adult households in which all adults employed outside of home (n = 754)**	264 (35.0%)	141 (18.7%)	N/A	59 (7.8%)	72 (9.6%)	23 (3.1%)	104 (13.8%)	31 (4.1%)	29 (3.9%)
**Number and age of children in household**									
**Households with 1 child (n = 943)**	188 (19.9%)	159 (16.9%)	375 (39.8%)	58 (6.2%)	N/A	26 (2.8%)	116 (12.3%)	27 (2.9%)	36 (3.8%)
**Households with >1 child (n = 1,283)**	278 (21.7%)	173 (13.5%)	492 (38.3%)	59 (4.6%)	136 (10.6%)	32 (2.5%)	143 (11.1%)	29 (2.3%)	52 (4.1%)
**Households with at least one child >12 yo (n = 1,126)**	189 (16.8%)	119 (10.6%)	375 (33.3%)	52 (4.6%)	141 (12.5%)	17 (1.5%)	244 (21.7%)	44 (3.9%)	47 (4.2%)
**Households in which all children are ≤12 yo (n = 1,103)**	239 (21.7%)	180 (16.3%)	476 (43.2%)	62 (5.7%)	18 (1.6%)	38 (3.5%)	12 (1.1%)	12 (1.1%)	37 (3.4%)

Households with an annual income of $25,000 or less most frequently reported that a non-working adult stayed home with the children (322 [45.4%]), whereas households with an annual income of at least $75,000 most frequently reported that an adult who works outside the home stayed home with the children (101 [33.4%]). Although households in which at least one child was older than 12 years of age most frequently reported choosing a non-working adult to stay home with the children (375 [33.3%]), these households also selected “child old enough to care for him/herself” more frequently than in any other household groups (244 [21.7%]) (Table 6).

During the school closure, 569 (25.5%) households reported that they incurred additional expenses related to childcare arrangements; 432 (75.9%) of these households reported the actual amount. The median expense was $150 per household, ranging from $6 to $2,500. The highest average daily cost was incurred when children were left home without supervision ($254; range, $30-$900) or taken to work ($228; range, $20-$1,000), and when a working adult stayed home ($224; range, $30-$1,500) (Table 7).

**Table 7:** Cost of alternative childcare options. Harrison County School District, Mississippi, August 2012.

Who provided childcare during the school closure	Households reported additional expenses for childcare n (%)	Average (median, range) daily cost of childcare arrangements*
**Adult household member who works outside the household**	116 (26.9%)	$224 ($150, $30-$1,500)
**Adult who does not live in the household**	115 (26.6%)	$207 ($120, $6-$1,500)
**Non-working adult household member**	103 (23.8%)	$191 ($188, $25-$800)
**Took children to work**	48 (11.1%)	$228 ($168, $20-$1,000)
**Older sibling**	40 (9.3%)	$199 (150, $50- $750)
**Childcare program**	27 (6.3%)	$161 (120, $35- $900)
**Child old enough to care for him/herself**	23 (5.3%)	$175 ($150, $50- $500)
**Children left home without supervision**	20 (4.6%)	$254 ($200, $30-$900)
**Adult working from home**	16 (3.7%)	$201 ($110, $50-$650)

* Calculated based on data from 432 households that reported actual amount of additional expenses. Overall, median expense was $150 (range $6-$2,500) per household.

**Fig 1:**
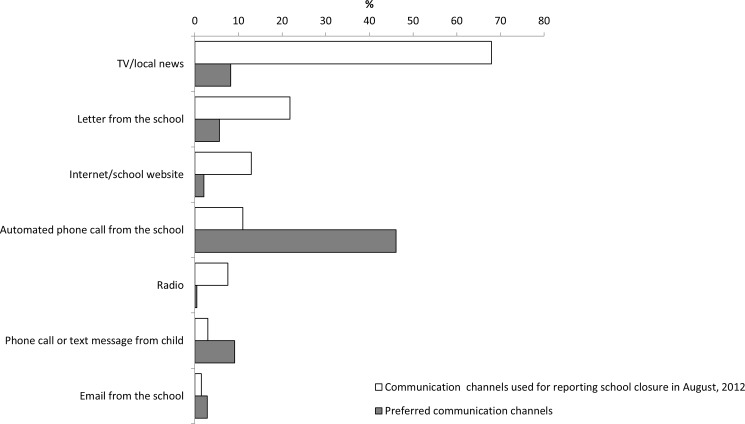
Communication of school closure information, Harrison County School District, Mississippi, August 2012.

The majority of respondents (1,514 [67.9%]) reported hearing about the unplanned school closure through television and local news outlets, followed by 486 (21.8%) respondents who received a letter from the school and 289 (12.9%) who learned about the closure from a school website. However, only 181 (8.2%) respondents reported that their preferred method of notification was through television or local news, while 1,018 (46.1%) indicated that an automated phone call from school was their preferred method of notification (Fig 1).

### Interviews with school officials

Twenty interviews were conducted with HCSD school officials: 14 with school principals, four with vice principals, and 2 with both the principal and vice principal. School officials in 19 (95%) interviews reported that the decision to close HCSD was made by the HCSD Superintendent. Information regarding the school closure was delivered to school officials directly from the Superintendent via email and walkie-talkie. All school officials reported having a protocol in place to help prepare for the possibility of an unplanned school closure, and that “doing so was second nature.” The majority of school officials reported sending letters home with students to announce the school closure and inform parents to watch the news and local media for information about school reopening dates. Most school officials, 14 (70%), thought that the communication strategy to families and school staff was effective and required minimal to no improvement. None of the schools had a plan in place for providing special education, meal replacement, continuing education, communication or childcare during school closure.

Sixteen (80%) school officials were concerned about the length of the school closure due to the need to complete make-up days, which are often taken from holiday vacation periods, when teachers and students have scheduled vacations and trips out of town. Scheduling make-up days during periods previously designated as holidays or vacation time resulted in high rates of absenteeism by students and staff and typically incurred additional financial costs. (Table 8)

**Table 8:** School administrator interviews: Major themes reported by principals and vice-principals (N = 20). Harrison County School District, Mississippi, August 2012.

Question	Major theme* (number of school administrators reporting theme)
**1. Please describe your official duties in relation to this school closure.**	Shelter duty (12) Secure school (11) Inform parents (7) Red Cross management (6) Inform teachers to secure classrooms (6)
**2. Who normally makes the decision to close schools in HCSD?**	Superintendent (19)
**3. What were your initial reactions and concerns about this school closure?**	Make-up days (9) Safety (5) Duration of school closure (4)
**4. Did you consult with other officials while making the decision to close this school?**	No (10) Yes, spoke with other principals (4) Yes, spoke with the superintendent or other district/county officials (4)
**5. Please describe the timeline during which the decision was made.**	24 hour lead time to prepare (5) Decision made over a few days (4)
**6. Was there a pre-planning process in place to help prepare for the possibility of an unexpected school closure?**	Yes (15) Pre-planning is “second nature” (7)
**7. How did you communicate this school closure to staff, parents, and students?**	Sent notice home to parents (16) Phone tree (13) Informed parents to watch the news/check website (13) Emailed staff and/or faculty meetings (13) Used school phone service messaging system to inform parents (7) Intercom announcement (6)
**8. What was effective about how you communicated this school closure to staff, parents, and students?**	Everything that was done (9) Informing parents to watch the news (6) Giving prior notice (6)
**9. What would you do differently next time in how you communicated this school closure to staff, parents, and students?**	Nothing (14)
**10. Did the school have a plan in place for each of the following while the school was closed (special education/therapy, meal replacement, continuing education, communication, childcare)? Please explain. If so, how was it communicated to staff and parents?**	No plan in place (18)
**11. How would each of the following change if the school was closed for more than one week (special education/therapy, meal replacement, continuing education, communication, childcare)?**	No, they would not change (11) Depends on available services (4) Messaging/calling system/iNow app (4)
**12. Were there any other major issues that your school faced due to this closure?**	No (11) Make-up day schedule (5) Concern over damage to school (4)
**13. In your opinion, what was done well?**	Communication (12) Timeline to school closure (4)
**14. In your opinion, what could be improved upon?**	Build in make-up days (4)

*Responses are not mutually exclusive

## Discussion

Our findings suggest that about half of households experienced at least some difficulty during the 4-day school closure in HCSD in Mississippi, most commonly uncertainty about duration of the school closure, difficulty arranging childcare, and lost pay. Almost half of the respondents noted that a month-long school closure, such as what may be needed during an influenza pandemic, would not be a major problem. The survey did not probe into the specific issues that would cause difficulties to parents and households due to hypothetical prolonged school closures.

The percentage of households reporting difficulty in our survey (almost 50%) is substantially higher than that reported to the randomized telephone poll during the 2009 Influenza A (H1N1) pandemic [8]. In that survey, 75% reported that the closure was not a problem at all and only 3% experienced major problems (compared with 25% in our survey). These differences can be explained by the shorter duration of the 2009 closures: 58% of respondents experienced a school closure that lasted 3 days or fewer.

Expectedly, one of the main challenges reported during this school closure was childcare. Arranging childcare was reported as difficult by a larger proportion of households in our survey (29%), compared with previous studies (in the Harvard poll [6], only 4% reported it as difficult). We found that if at least one child in the household is older than age 12, the household is about 50% less likely to report difficulty with childcare. Similarly, the North Carolina study [14], which had a higher proportion of children age 12 years and older than our survey population, reported a smaller proportion of parents missing work. According to the US Census, in general, the proportion of children in self-care in the United States is much higher in 12–14 year olds (26.9%) compared to 5–11 year olds (4.7%) [15]. Thus, in case of the prolonged closures, such as those that can be implemented during severe pandemic, some of the preparedness efforts would need to be directed to providing alternative childcare arrangements for younger children. In our survey, childcare programs were rarely chosen, regardless of income or family composition. Perhaps such programs are unavailable in the HCSD area, but exploring the factors that may influence this trend was outside the scope of our survey.

Childcare decisions varied by family composition and adult employment; working single parents most frequently used outside help, whereas in households with two or more adults who all work outside of home, the number one choice was for a working adult to stay home with the children. Similar to previously evaluated school closures, the first choice for alternative childcare across all households was “non-working adult household member” [5]. Households with all adults employed outside the home were three times more likely to report difficulty with childcare arrangements compared with households where a non-working adult was available to provide childcare. Moreover, adults employed outside the home were almost three times more likely to miss work or lose pay during the school closure, compared to those with alternative employment arrangements (e.g., adults working from home).

The proportion of households reporting missed work and lost pay in our survey was much higher than in previous studies [13–15]; in part, this could be related to workplace closures that were implemented in the area as a hurricane preparedness measure. Also, availability of a non-working adult who could stay home with children played an important role in employment interruption. In a North Carolina study [14], for example, the number of employed adults was very similar to that in HCSD (54%); however, unlike in HCSD, the majority of households in North Carolina had at least one non-working adult available to stay home with the children. We have also demonstrated that if adults stayed home from work longer, they are more prone to losing pay. In the United States, the proportions of families in which both parents in married-couple households with children and adults in single-parent households are employed is relatively high (59% and 67–81%, respectively)[16]. This proportions are similar to that in our survey: in 45% of households with two or more adults, all adults were employed outside of home, and 75% of single-adult households, had the adult employed outside. Because parental participation in the work force is high and continues to grow, interruption of employment during school closures may cause financial concerns (in our survey, loss of pay during the closure was the second most reported cause for perceived difficulty). It is important for policy makers to address paid leaves, work-from-home options, and alternative childcare provision, especially in communities with a high proportion of young children who cannot stay home alone while schools are closed.

The cost of arranging alternate childcare ranged widely ($6-$2,500), which might be a function of the way the question was formulated and/or understood. The survey question asked respondents to consider costs related to childcare, food, gas, and other needs but did not ask for item-specific estimates, which might have created discrepancies in the responses. Only a small percentage of HCSD households reported challenges related to missed school-provided meals, despite high district enrollment in the program. This can, in part, be explained by the short duration of the school closure. Similarly, in previous surveys, missing free or reduced-price lunch was reported as a major problem by only < 1%-10% of families [5,6].

We did not inquire how families would cope with the lack of subsidized school-based meals; however, planning for emergency food distribution during prolonged school closures may be particularly important for families who appear to be most vulnerable to this problem (such as families with 3 or more children, as determined by our survey). During the 2009 H1N1 pandemic, USDA operated a Pandemic Supplemental Nutrition Assistance Program (P-SNAP) to provide food to children eligible for free or reduced-price school lunches whose schools are closed for at least 5 consecutive days during a pandemic emergency designation [17]. Similar measures should be considered during school closures due to other types of emergencies on a local or state level. When resources for supplementary food supplies are scarce, school administrators and other officials can consider triaging eligible populations by vulnerability level.

Despite the effort school officials undertook to inform families (e.g. letters sent home with students) about the unplanned school closure, the majority of parents learned about the closure through television and local news outlets. Many parents indicated that in case of emergency, their most preferred way to be notified is by automated phone call from the school. School officials should work with parents to address these communication issues when planning for future emergencies.

Our survey is subject to several limitations. First, our sample provides data on only 28.4% of the households with students in HCSD schools. However, compared with similar surveys completed by only 200–500 households [11–16], our sample size is large and the socio-demographic characteristics of household respondents are similar in terms of income, education, race, and family size [8]. Therefore, we believe our findings may be applied to all HCSD households.

Second, the survey questionnaire may not have included all possible consequences resulting from this school closure, although we pilot-tested the survey prior to implementing the investigation to maximize the appropriateness and comprehensibility of the questions.

Third, the timing of the survey (distributed 3 months after the school closure) may have detracted from the accuracy of the responses. It is also possible that families that experienced difficulties during the closure were more likely to respond to the questionnaire than those who experienced few or no difficulties.

Fourth, the letter accompanying the survey explained that only one survey questionnaire is requested from each household, regardless of the number of children in the household enrolled in HCSD. Therefore, a possibility that some households submitted more than one filled questionnaire is low.

Fifth, while we did request that respondents report additional expenses for childcare, we did not ask that the expenses be itemized, so they were reported as crude numbers, which may not be completely accurate.

Lastly, this unplanned school closure was implemented in anticipation of Hurricane Isaac, so our findings may not fully reflect unplanned school closures during influenza pandemics. However, school closures in Harrison County were implemented as a preparedness measure, and no damages to school facilities and surrounding communities were made by the storm. Therefore, school closures discussed in this report can serve as a reasonable proxy to an influenza pandemic situation.

## Conclusions

While planning to close schools during an influenza pandemic or other emergency, school and public health authorities should account for the unintended consequences that school closures can have for student families. Certain characteristics of the source population should be evaluated in order to better target policy decisions to local needs. These may include the proportion of small children who cannot be left home without supervision, presence of multiple children in the household, employment of all household adults outside the home, household income below the poverty level, and proportion of children eligible for subsidized school meals.

In our survey of a large rural fringe school district, finding alternative childcare was particularly disruptive for households in which all adults work outside of home, making them highly likely to miss work or lose pay. Introducing paid leave or remote work options could safeguard working parents’ employment and income during school closures and should be strongly considered in preparedness planning discussions. Availability of alternative childcare can protect families with small children from employment insecurities and child safety issues, particularly those families in which all adults work outside of home.

Provision of food to households participating in the National School Lunch Program in the absence of subsidized school meals during school closures should be included in preparedness plans. When necessary (e.g. if the resources are scarce), the most vulnerable groups should be identified and given priority during local food distribution.

Emergency communication strategies to alert parents about future unplanned school closures should also be reviewed to ensure that parents receive timely notification through preferred communication channels, which may allow families extra time to prepare for school closures. Additional evaluations of unplanned school closures implemented in other communities will help determining whether these findings are applicable in other geographic locations or socio-economic groups.

## Supporting information

S1 Database (XLSX)

S1 Data Dictionary (XLSX)

S1 Survey Administrators. (DOCX)

S2 Survey Parents. (DOCX)
