# Supplementary material for: Unintended costs and consequences of school closures implemented in preparation for Hurricane Isaac in Harrison County School District, Mississippi, August-September 2012
Source: PLoS One. 2017 Nov 1;12(11):e0184326. doi: 10.1371/journal.pone.0184326 (PMC5665504; doi:10.1371/journal.pone.0184326)
Supplement: S1 Survey — (DOCX) [file pone.0184326.s003.docx]

**School Administrator Survey**

The Centers for Disease Control and Prevention (CDC) is partnering with Mississippi Department of Health and local public health authorities to evaluate the consequences of unplanned school closures. Information from this survey will help Mississippi better prepare for such potential closures during public health emergencies. Also, this survey will help inform CDC’s existing recommendations related to school closures, as well as help state and local public health authorities learn more about the experiences school officials face during unexpected school closures.

This is an open-ended survey for school administrators who were involved in the recent Harrison County school closure that occurred in late August 2012 due to a preparation of Hurricane Isaac. We are trying to learn more about the experiences school officials faced, as well as, get more information on the needs and concerns from school officials during extended unplanned school closures like this one.

Your participation is voluntary, and you can skip any questions that you would prefer not to answer. Anything you tell us will be kept **CONFIDENTIAL** and shared only with the CDC, as well as your state and local public health authorities.

**Your response to this survey indicates that you agree to participate in this investigation and agree to have your responses tape recorded.**

Thank you for your participation in this important project.

School name_____________________________________________________________

Contact Phone #/E-mail address _____________________________________________

Position title at time of closure decision________________________________________

Years in position at time of closure ___________________________________________

1. Please describe your official duties in relation to this school closure:
2. Who normally makes the decision to close schools in Harrison County School District?
3. What were your initial reactions and concerns about this school closure?
4. Did you consult with other officials while making the decision to close this school?
5. Please describe the timeline during which the decision was made.
6. Was there a pre-planning process in place to help prepare for the possibility of an unexpected school closure?
7. How did you communicate this school closure to staff, parents, and students?
8. What was effective about how you communicated this school closure to staff, parents, and students?
9. What would you do differently next time in how you communicated this school closure to staff, parents, and students?

**Please consider some of the major issues as a result of this school closure.**

1. Did the school have a plan in place for each of the following while the school was closed? Please explain. If so, how was it communicated to staff and parents?

Special education/therapy?

Meal Replacement?

Continuing Education?

Communication?

Childcare?

1. How would each of the following change if the school was closed for more than one week?

Special education/therapy?

Meal replacement?

Continuing education?

Communication?

Childcare?

1. Were there any other major issues that your school faced due to this school closure?
2. In your opinion, what was done well?
3. In your opinion, what could be improved upon?
