# Supplementary material for: Unintended costs and consequences of school closures implemented in preparation for Hurricane Isaac in Harrison County School District, Mississippi, August-September 2012
Source: PLoS One. 2017 Nov 1;12(11):e0184326. doi: 10.1371/journal.pone.0184326 (PMC5665504; doi:10.1371/journal.pone.0184326)
Supplement: S2 Survey — (DOCX) [file pone.0184326.s004.docx]

#

Dear Parents,

As you know, the school that your child is attending was closed on August 28th, 2012 due to preparation for the Hurricane Isaac. The Centers for Disease Control and Prevention (CDC) is partnering with the Mississippi Department of Health, Harrison County School District and to learn more about the effects the school closure had on children and their families. Information from this survey will help us evaluate the cost and consequences of unplanned school closures and better prepare for future potential closures during public health emergencies. This information is also important to you because it will help us and the school administration to learn about the difficulties that families face when schools are closed unexpectedly, to understand how we can better respond to future unexpected closings, and to improve communication between families and the school.

This is a survey for you as a parent or guardian of a child attending a school within the Harrison County School District. The survey is anonymous and your participation is voluntary. It will take no more than 15 minutes to complete. Anything you tell us will be kept **CONFIDENTIAL** and shared only with the CDC, and the state health and school authorities.

**Your response to this survey indicates that you agree to participate in this investigation.**

**Please fill out only one questionnaire per household (even if you have more than 1 child attending the same school) and return it to your child’s school from which this survey was sent by November 30^th^.**

Thank you for your participation in this important project!

**Let’s begin by listing all of the children in your home. Please identify children who live in your household that attend school (PK-12):**

| **Subject  ID** | **Gender (M/F)** | **What is your relationship to him/her?** | **Grade** | **School name** |
| --- | --- | --- | --- | --- |
| Child- 1 |  |  |  |  |
| Child -2 |  |  |  |  |
| Child -3 |  |  |  |  |
| Child -4 |  |  |  |  |
| Child -5 |  |  |  |  |

**Now, we would like to ask you about your child’s or children’s** **school closure.**

1. **How did you learn about the closure (mark all that apply)?**

**** Automated phone call from the school

**** Phone call or text message from your child

**** Email from the school

 Letter from the school

**** Internet/School’s website

**** Radio

**** TV/local news

**** Other **________________________________________________________________**

**** Don’t remember/not sure

1. **In case of future emergencies, what is your most preferred way to be contacted?**

**** Automated phone call from the school

**** Phone call or text message from your child

 Email from the school

 Letter from the school

 Internet/School’s website

**** Radio

**** TV/local news

 Other ________________________________________________________________

 Don’t know/ not sure

**Now, we would like to ask you some questions about** **childcare (babysitters, daycare facility, etc.)**

1. **What did you do for childcare while the schools were closed? (Mark all that apply)**

□ Non-working adult household member or homemaker

□ Working adult household member who works from home

□ Working adult household member who works outside the household

□ Older sibling less than 18 years of age

□ Adult who does not live in the household

□ Childcare program

□ Child(ren) home without supervision

□ Took child(ren) to work

□ Nothing, is not needed due to age of child(ren)

□ Other *(specify):___________________________________________________________*

□ Prefer not to answer

1. **While the school was closed, what places did your child(ren) go to and what activities did he or she participate in? Please fill this out for each child in your household.**

|  | Child 1 | Child 2 | Child 3 | Child 4 | Child 5 |
| --- | --- | --- | --- | --- | --- |
| 1.Library | □ Yes □ No | □ Yes □ No | □ Yes □ No | □ Yes □ No | □ Yes □ No |
| 2.Art center | □ Yes □ No | □ Yes □ No | □ Yes □ No | □ Yes □ No | □ Yes □ No |
| 3.Sports activities (such as  practices/games/events, roller rink, 4 wheeling) | □ Yes □ No | □ Yes □ No | □ Yes □ No | □ Yes □ No | □ Yes □ No |
| 4. Public gatherings (such as concerts, movies, festivals). Please specify what event | □ Yes □ No  *Please specify*  *______________* | □ Yes □ No  *Please specify*  *______________* | □ Yes □ No  *Please specify*  *______________* | □ Yes □ No  *Please specify*  *____________* | □ Yes □ No  *Please specify*  *____________* |
| 5. Religious services | □ Yes □ No | □ Yes □ No | □ Yes □ No | □ Yes □ No | □ Yes □ No |
| 6. Grocery shopping | □ Yes □ No | □ Yes □ No | □ Yes □ No | □ Yes □ No | □ Yes □ No |
| 7. Strip Malls / Walmart | □ Yes □ No | □ Yes □ No | □ Yes □ No | □ Yes □ No | □ Yes □ No |
| 8. Restaurants | □ Yes □ No | □ Yes □ No | □ Yes □ No | □ Yes □ No | □ Yes □ No |
| 9. Part-time job  If Yes, list employer: | □ Yes □ No | □ Yes □ No | □ Yes □ No | □ Yes □ No | □ Yes □ No |
| 10.Friends’ houses, or have  friends at child’s own house  If Yes, what was the maximum  number of people present? | □ Yes □ No  Maximum #  present: | □ Yes □ No  Maximum #  present: | □ Yes □ No  Maximum #  present: | □ Yes □ No  Maximum #  present: | □ Yes □ No  Maximum #  present: |
| 11. Visit family  If Yes, what was the maximum  number of people present? | □ Yes □ No  Maximum # present: | □ Yes □ No  Maximum # present: | □ Yes □ No  Maximum # present: | □ Yes □ No  Maximum # present: | □ Yes □ No  Maximum # present: |
| 12. Other, please list:_____________________ | □ Yes □ No | □ Yes □ No | □ Yes □ No | □ Yes □ No | □ Yes □ No |

1. **Did the school provide recommendations for alternative childcare arrangements during the closure?**

□ Yes. What was recommended?_____________________________________________

□ No

□ Don’t know / not sure

1. **Did you have additional expenses for childcare arrangements because schools were closed for one week?**

□ Yes, How much? (please consider childcare, food, gas, & other cost)________________

□ No

**Now, identify each adult (Adult-1-Adult-5) 18 years and older, including yourself, who lives in your home.**

| **Subject  ID** | **Gender**  **(M or F)** | **Age** | **Is he/she a parent or guardian of a child in the home?** | **Is he/she a major wage earner in the home?** |
| --- | --- | --- | --- | --- |
| Adult -1 |  |  | □ Yes □ No |  Yes  No |
| Adult -2 |  |  | □ Yes □ No |  Yes  No |
| Adult -3 |  |  | □ Yes □ No |  Yes  No |
| Adult -4 |  |  | □ Yes □ No | Yes  No |
| Adult -5 |  |  | □ Yes □ No | Yes  No |

**Thank you again for your time, the information that you are providing is extremely helpful. You are half way through the survey.**

1. **We would like to ask some questions about the effect the school closures had on you and the other adults in your household. Please answer each question for Adult-1 to Adult-5.**

| **Is the adult household member employed outside of the household?** | **Adult 1**  □ Yes □ No  □ N/A | **Adult 2**  □ Yes □ No  □ N/A | **Adult 3**  □ Yes □ No  □ N/A | **Adult 4**  □ Yes □ No  □ N/A | **Adult 5**  □ Yes □ No  □ N/A |
| --- | --- | --- | --- | --- | --- |
| **For the adults listed, what are the** **hours worked per week?** | □ Full-time  □ Part-time  □ No fixed schedule  □ N/A | □ Full-time  □ Part-time  □ No fixed schedule  □ N/A | □ Full-time  □ Part-time  □ No fixed schedule  □ N/A | □ Full-time  □ Part-time  □ No fixed schedule  □ N/A | □ Full-time  □ Part-time  □ No fixed schedule  □ N/A |
| **For the adults listed, do they have the option to work from home?** | □ Yes □ No  □ Sometimes  □ Don’t know | □ Yes □ No  □ Sometimes  □ Don’t know | □ Yes □ No  □ Sometimes  □ Don’t know | □ Yes □ No  □ Sometimes  □ Don’t know | □ Yes □ No  □ Sometimes  □ Don’t know |
| **For the adults listed, do they receive paid time off (sick and/or annual leave)?** | □ Yes □ No  □ Don’t know | □ Yes □ No  □ Don’t know | □ Yes □ No  □ Don’t know | □ Yes □ No  □ Don’t know | □ Yes □ No  □ Don’t know |
| **For the adults listed, did their employer allow them to take time off during the school closure?** | □ Yes □ No  □ Don’t know | □ Yes □ No  □ Don’t know | □ Yes □ No  □ Don’t know | □ Yes □ No  □ Don’t know | □ Yes □ No  □ Don’t know |
| **For the adults listed, Did they** **miss any work while schools were closed?** | □ Yes □ No  □ Don’t know | □ Yes □ No  □ Don’t know | □ Yes □ No  □ Don’t know | □ Yes □ No  □ Don’t know | □ Yes □ No  □ Don’t know |
| **If yes, how many days of work time were lost?** | □ < 1 day □ 1-2 days □ 3-5 days □ > 1 week  □ Don’t know | □ < 1 day □ 1-2 days □ 3-5 days □ > 1 week  □ Don’t know | □ < 1 day □ 1-2 days □ 3-5 days □ > 1 week  □ Don’t know | □ < 1 day □ 1-2 days □ 3-5 days □ > 1 week  □ Don’t know | □ < 1 day □ 1-2 days □ 3-5 days □ > 1 week  □ Don’t know |
| **For the adults listed, did they lose pay during this time off?** | □ Yes □ No  □ Don’t know | □ Yes □ No  □ Don’t know | □ Yes □ No  □ Don’t know | □ Yes □ No  □ Don’t know | □ Yes □ No  □ Don’t know |

1. **Did the recent school closure cause difficulty for you to provide food for your family because of the loss of meals provided by the Free and Reduced School Breakfast/Lunch program?**

 Yes

 No

 My child(ren) is not enrolled in the free and reduced lunch program

 Prefer not to answer

1. **During the school closure, did you receive information from your school about how you can get breakfast or lunch meals for your child(ren)?**

 Yes, How did you receive the information?___________________________________

 No

 Don’t know / not sure

1. **If your child(ren) receive(s) special education and/or related support services (students with disabilities) at the school, did school officials provide you with other options for these services for your child(ren)?**

 Yes, How did they provide the information?___________________________________

 No, Were you able to provide these services?_______If yes, how much did it cost?_____

 My child(ren) do(es) not receive special education and/or related support services

 Don’t know / not sure

1. **When the school was closed, were school officials able to provide any alternative options for continuing education, such as reading material or internet courses?**

 Yes, How did they provide these other options ?_________________________________

 No,

 Don’t know / not sure

1. **If this school closure was difficult for you and your family,** **what were some things that made the situation difficult? (check all that apply)**

□ Difficult to make childcare arrangements

□ Expensive to make childcare arrangements

□ Lost income due to missed work

□ Student missed school meals

□ Uncertainty about how long school would be closed

□ Other (please describe _______________________)

□ I did not find this school closure being difficult for me and my family

1. **If schools or childcare facilities had to be closed for ONE MONTH, how big of a problem would it be for you to take care of the children in your household for that long?**

□ Major problem,Why?___________________________________________________

□ Medium/Moderate problem,Why?_________________________________________

□ Minor problem,Why?___________________________________________________

□ Not a problem

□ Don’t know/ Not sure

1. **If schools or childcare facilities were closed for ONE MONTH, who would mainly take care of the child(ren) who live in your household**? **(**check all that apply**)**

□ Parent(s) □Another family member who lives in home

□ Teenage family member □ Babysitter or “nanny”

□ A family member who lives outside home □ Friends

□ Neighbors □ Children would take care of themselves

□ Other (please describe _______________________)

□ Don’t know/ not sure

1. **What is your race/ethnicity?**

□ White Non-Hispanic

□ Black/African American Non-Hispanic

□ Hispanic/Latino

□ Native American Indian/Alaska Native

□ Native Hawaiian/Pacific Islander

□ Asian

□ Other _________________________

1. **What is the annual household income from all sources?**

□ Less than $15,000

□ $15,000 to $24,999

□ $25,000 to $34,999

□ $35,000 to $49,999

□ $50,000 to $74,999

□ Greater than or equal to $75,000

□ Prefer not to answer

1. **What is the highest grade or year of school that the primary care giver/guardian has completed?**

□ Grades 1-8

□ Grades 9-11

□ Grade 12 or GED (high school graduate)

□ College: 1-3 years or technical school training

□ College: 4 years or more (college graduate)

□ Graduate or professional school: (1 year or more)

□ Other (specify)________________________________________________________

□ Prefer not to answer.

**We would like to know about your experience in filling out this survey. Please rank the survey from 1-5, with 1 being the most difficult to understand to 5 being easy to understand.________**

**Thank you for your time!**
